# Evaluation of a prototype biplex VIDAS immunoassay for type-specific detection of HSV-1 and HSV-2 IgG antibodies

**DOI:** 10.1128/jcm.00931-26

**Published:** 2026-09-30

**Authors:** Marie Roccon, Hardy Charly, Aurélie Truffot, Laurent Pelletier, Patrice Morand, Olivier Epaulard, Raphaële Germi, Julien Lupo

**Affiliations:** 1 Laboratoire de Virologie, Université Grenoble Alpes, CHU Grenoble Alpes 36724 , Grenoble, France; 2 Laboratoire de Virologie, Université Grenoble Alpes, CEA, CNRS, Institut de Biologie Structurale (IBS), CHU Grenoble Alpes 36724 , Grenoble, France; 3 Service d’Infectiologie, Université Grenoble Alpes, CEA, CNRS, Institut de Biologie Structurale (IBS), CHU Grenoble Alpes 36724 , Grenoble, France; St Jude Children's Research Hospital, Memphis, Tennessee, USA

**Keywords:** herpes simplex virus, HSV-1, HSV-2, serology, immunoassay, VIDAS, LIAISON

## Abstract

Type-specific serologic detection of IgG antibodies to herpes simplex virus (HSV)-1 and HSV-2 provides important diagnostic and epidemiological information. Novel assays are under development and require rigorous evaluation before routine clinical implementation. We compared the VIDAS HSV 1&2 IgG prototype assay (bioMérieux) with the widely used LIAISON HSV-1 and HSV-2 IgG assays (DiaSorin). Western blot (WB) analysis was used as the reference method to resolve discrepant results. A total of 501 serum samples from 348 patients were analyzed, including 259 patients with PCR-confirmed HSV-1 or HSV-2 infection. WB was performed on the 89 discordant samples between the two assays. For HSV-1 IgG detection, the VIDAS prototype assay showed a sensitivity of 95.4% and a specificity of 98.6%, compared with 98.9% and 94.9%, respectively, for the LIAISON HSV-1 IgG assay. For HSV-2 IgG, the VIDAS assay demonstrated a sensitivity of 100% but a lower specificity of 90.7%, whereas the LIAISON HSV-2 IgG assay showed a lower sensitivity of 88.2% and a higher specificity of 99.4%. Approximately 30% of the samples with high HSV-1 IgG index values yielded false-positive HSV-2 IgG results with the VIDAS assay. The receiver operating characteristic curve analysis indicated that increasing the VIDAS HSV-2 positivity threshold from 0.063 to 0.137 improved specificity to 98.9% while maintaining a sensitivity of 95.4%. For HSV-1 IgG detection, the VIDAS prototype assay showed performance comparable to that of the LIAISON assay. For HSV-2 IgG, optimization of the positivity threshold may substantially improve specificity, an important consideration in low-prevalence populations.

**IMPORTANCE:**

Type-specific serology is an important complement to molecular diagnosis of herpes simplex virus (HSV) infections, helping distinguish primary, recurrent, and non-primary infections; assess neonatal transmission risk; and support patient counseling. Because western blot, the reference method, is not routinely available, the clinical value of HSV serology relies on the performance of automated immunoassays. We evaluated a novel VIDAS HSV 1&2 IgG biplex prototype assay and compared it with a widely used commercial assay. The VIDAS assay showed performance comparable to the LIAISON assay for HSV-1 IgG detection while offering simultaneous detection of HSV-1 and HSV-2 antibodies in a single test. For HSV-2 IgG, however, false-positive results were observed, particularly in samples with high HSV-1 antibody levels. Importantly, optimizing the HSV-2 positivity threshold improved specificity while maintaining high sensitivity. These findings provide data for assay optimization before commercialization and highlight the need to balance sensitivity and specificity for accurate clinical interpretation.

## INTRODUCTION

Herpes simplex viruses (HSV) types 1 and 2 belong to the *Herpesviridae* family and establish lifelong persistent infections in their hosts. Following a primary infection, often asymptomatic, HSV remains latent in the nuclei of sensory ganglion cells and may periodically reactivate. HSV infections are highly prevalent and generally mild; however, they affect billions of individuals worldwide and remain a significant cause of morbidity. Certain clinical manifestations can be severe and life-threatening without prompt diagnosis and treatment, notably herpes encephalitis and neonatal herpes (1, 2).

Although the clinical relevance of distinguishing between HSV-1 and HSV-2 is sometimes debated, these two viruses exhibit distinct clinical and epidemiological characteristics. HSV-1 is typically associated with oral lesions but is increasingly recognized as a cause of genital herpes, whereas HSV-2 is classically linked to genital infections (3, 4). Recurrences and subclinical viral shedding are considerably more frequent in genital HSV-2 infection than in genital HSV-1 infection (5). Consequently, viral typing provides clinically relevant information, particularly regarding the expected frequency of recurrences, and supports appropriate patient counseling (6, 7). Pre-existing HSV-2 infection may confer only partial protection against acquisition of HSV-1, whereas prior HSV-1 infection may reduce the likelihood of symptomatic HSV-2 infection (8). According to recent epidemiological data from France, approximately 67% of individuals under 50 years of age are HSV-1 seropositive, while HSV-2 seroprevalence in the same age group is estimated at around 16% (9).

The method of choice for diagnosing herpetic disease is the detection of HSV-1 or HSV-2 DNA by PCR from mucocutaneous samples or from deeper specimens, such as bronchoalveolar lavage fluid, cerebrospinal fluid, or blood, depending on the clinical presentation. PCR is well suited for the diagnosis of active infection but has limited value in asymptomatic individuals (7). Serological testing, particularly type-specific assays that detect and differentiate IgG antibodies against HSV-1 and HSV-2 (glycoprotein G1 for HSV-1 and glycoprotein G2 for HSV-2), provides complementary information, enabling the classification of infection as a primary, recurrent, or non-primary initial (i.e., infection with one HSV type in an individual previously exposed to the other type) (6). Clinical manifestations vary across these stages, with generally milder symptoms and reduced, shorter-duration viral shedding during recurrences compared with primary infection (10). Type-specific serology is of particular clinical value in pregnant women experiencing a first recognized episode of genital herpes, as it helps assess the risk of neonatal transmission. This risk is estimated at 30%–50% in cases of primary maternal infection, approximately 25% in non-primary infection, and less than 5% in recurrent disease (11). Although PCR remains the reference method for diagnosing genital herpetic lesions, type-specific IgG testing provides additional information that may help guide clinical management, particularly in the context of neonatal HSV infection. Current guidelines therefore recommend the use of type-specific HSV serology during pregnancy, while discouraging non-type-specific assays and routine screening (7, 11). Type-specific HSV serology may also be useful in sexual partners of individuals with a known HSV infection for counseling on transmission risk (7). Its role in the transplantation setting is more debatable, as all recipients receive appropriate prophylaxis to prevent HSV infection regardless of their serostatus (12–15).

The reference standard in HSV serology is western blot (WB) analysis, which offers high sensitivity and specificity through the detection of antibodies directed against multiple HSV-1 and HSV-2 proteins (16–18). However, this method is labor-intensive, costly, and available only in a limited number of specialized laboratories. Automated immunoassays have therefore been developed to improve accessibility and enable high-throughput testing; however, their performance varies considerably, highlighting the need to conduct comparative evaluations of the available platforms. bioMérieux has recently developed a prototype VIDAS serological assay capable of detecting and distinguishing IgG antibodies against HSV-1 and HSV-2. To date, no data have been published on the performance of this prototype, which is intended for potential commercial use.

The aim of this study was to compare the analytical performance of the VIDAS HSV 1&2 IgG prototype assay with that of the LIAISON HSV-1 and HSV-2 IgG assays, using WB analysis as the reference method to resolve discordant results. The study included samples from both asymptomatic and symptomatic patients with PCR-confirmed active herpetic infection.

## MATERIALS AND METHODS

### Study design and sample collection

The study was conducted between June and August 2025 in the virology laboratory at Grenoble Alpes University Hospital (CHUGA). The cohort comprised serum samples collected retrospectively from non-transplant patients with active herpetic infection, as evidenced by the detection of HSV-1 or HSV-2 DNA by PCR in various specimens (including mucocutaneous and genital lesions, cerebrospinal fluid, blood, bronchoalveolar lavage, and ocular specimens) between 2024 and 2025. These serum samples were obtained in the weeks preceding or following the positive PCR result. To obtain samples from HSV-seronegative patients, we selected specimens that had previously tested negative for HSV-1/2 IgG using the LIAISON HSV-1/2 non-specific IgG assay. To increase the number of samples positive for anti-HSV-2 IgG, additional serum specimens were prospectively collected between June and August 2025 and screened using the LIAISON HSV-2 type-specific IgG assay (Fig. S1). The study was approved by the Directorate for Scientific Research at CHUGA (reference number 38RC25.0207). All patients included in the study were informed about it and did not object.

### Assay comparison

The samples were tested using two assays: the DiaSorin LIAISON HSV-1 and HSV-2 IgG assays (DiaSorin, Saluggia, Italy), and the bioMérieux prototype VIDAS HSV 1&2 IgG assay (bioMérieux, Marcy l’Etoile, France). The LIAISON platform provides two independent assays, whereas the VIDAS prototype enables simultaneous detection of HSV-1 and HSV-2 IgG in a single biplex test. Samples with equivocal results on the DiaSorin LIAISON assay, samples with index values close to the positivity threshold on the bioMérieux VIDAS assay (index 0.040–0.048 for HSV-1 IgG and 0.060–0.066 for HSV-2 IgG), and samples with discordant results between the two assays were further investigated by WB analysis. The reference result was defined as the initial serological result when the two assays were concordant. In cases of discordance, the WB result was used as the reference, supported, when necessary, by PCR findings, serological follow-up, and relevant clinical information. To avoid repeated freeze-thaw cycles, each serum sample was tested using both assays, and when required, WB, within the same week. Each serum sample underwent a maximum of one freeze-thaw cycle.

### DiaSorin LIAISON HSV-1 and HSV-2 IgG assays

The LIAISON HSV-1 or HSV-2 IgG assays are automated chemiluminescent immunoassays that detect IgG antibodies against HSV-1 gG1 or HSV-2 gG2 glycoproteins, respectively. Tests were performed on the LIAISON XL platform in accordance with the manufacturer’s instructions. The results were expressed as index values relative to the calibrators. Values <0.9 were considered negative; between 0.9 and 1.1, equivocal; and ≥1.1, positive. In this study, the reported index values ranged from <0.01 to >62.2 for the HSV-1 assay and from 0.5 to >15 for the HSV-2 assay. Quality control (QC) and calibration were performed for each assay according to the manufacturer’s instructions.

### bioMérieux prototype VIDAS HSV 1&2 IgG assay

The VIDAS HSV 1&2 IgG prototype assay is a qualitative, fully automated immunoassay that is based on the principle of an enzyme-linked fluorescent assay. This assay can detect and differentiate simultaneously IgG directed against HSV-1 and HSV-2 in a single run (biplex test). Analyses were performed using VIDAS KUBE instruments. Each run includes the S1 standard, which is tested in duplicate, as well as a positive control (C1). Relative fluorescence values (RFV) are recorded, and index values are calculated according to the manufacturer’s instructions. HSV-1 index = RFV4 (sample or control)/mean RFV4 (standard S1). HSV-2 index = RFV2 (sample or control)/mean RFV2 (standard S1). According to the manufacturer’s instructions, HSV-1 index values ≥ 0.044 (corresponding to RFV4 values ≥ 50) and HSV-2 index values ≥ 0.063 (corresponding to RFV2 values ≥ 110) were considered positive; index values below these thresholds were considered negative. The manufacturer did not define an equivocal zone. Standard performance was monitored in accordance with the following acceptance criteria: coefficients of variation ≤5% for RFV2 and ≤10% for RFV4.

### Western blot analysis

The Anti-HSV-1/HSV-2-gG2-EUROLINE-WB (IgG) western blot assay (EUROIMMUN, Lübeck, Germany) was used to determine the presence of specific antibodies against HSV-1 and HSV-2 simultaneously. HSV-1 status is determined by reactivity to the gC1 and gG1 antigens, while HSV-2 status is determined by reactivity to the gG2 antigen. WB reading is automated using Euroline Scan software. The software automatically records and identifies the bands and measures their intensity. In addition, two independent operators performed a visual reading of the WB. Profiles that were indeterminate or discordant between the two operators were excluded from the statistical analysis.

### Statistical analysis

The sensitivity, specificity, positive predictive value (PPV), negative predictive value (NPV), and Cohen’s kappa were all calculated with 95% confidence intervals (CI). Equivocal results obtained with the LIAISON assays were considered positive for calculating specificity and negative for calculating sensitivity. Samples showing discordant results that could not be resolved by WB were classified as indeterminate and excluded from the statistical analysis. When multiple samples from the same patient were available, all were included and analyzed as independent samples. A supplementary performance analysis (sensitivity and specificity) was performed using one serum sample per patient, preferentially selecting the sample collected at the time of PCR testing or, when unavailable, the earliest available sample. The PPV and NPV were adjusted using estimated HSV-2 and HSV-1 prevalence rates of 15% and 60%, respectively, in the French population. Cutoff points for IgG levels were optimized using receiver operating characteristic (ROC) curve analysis and the Youden index. Sensitivity and specificity, as well as PPV and NPV values, were compared between the VIDAS and LIAISON assays using the McNemar test or a two-sided Z test, respectively. Agreement between the assays and the reference result was assessed using Cohen’s kappa coefficient. Differences between kappa coefficients were evaluated using a two-sided Z test. *P* values <0.05 were considered significant. All statistical analyses were conducted using GraphPad Prism software (version 9.2.0, GraphPad Software, San Diego, CA, USA) and RStudio.

## RESULTS

### Study population

A total of 510 serum samples were initially collected. Seven samples were excluded due to insufficient volume to complete all analyses. Two samples from transplant patients were also excluded subsequently. The final data set comprised 501 serum samples from 348 patients. The clinical characteristics of the patients are summarized in Table S1. Of the 323 samples that tested positive for HSV-1 or HSV-2 IgG, 259 (80%) were obtained from patients with PCR-confirmed HSV-1 or HSV-2 infection. Of the 270 patients in our cohort who were tested for HIV, 13 were HIV-positive. All of these patients were infected with HSV-2: nine were positive for both HSV-1 and HSV-2 IgG, and four were positive for HSV-2 IgG and negative for HSV-1 IgG. WB analyses were conducted on the 89 discordant results between the LIAISON and the VIDAS assays. Final HSV status was determined based on WB and, when necessary and available, supported by a review of the clinical, PCR, and serological history. Ultimately, 167 samples were classified as HSV-1 IgG-positive and HSV-2 IgG-negative, 36 as HSV-1 IgG-negative and HSV-2 IgG-positive, 114 as positive for both HSV-1 and HSV-2, and 177 as negative for both viruses and 7 as indeterminate for HSV status (flow chart of the study is provided in Fig. S1).

### Assay performance for the detection of anti-HSV-1 IgG

The analytical performance of the VIDAS HSV 1&2 IgG prototype assay and LIAISON HSV-1 assays is presented in Table 1. The VIDAS assay demonstrated a sensitivity of 95.4% and a specificity of 98.9%. The LIAISON assay had the highest sensitivity (98.6%), but lower specificity (94.9%). Both assays showed excellent concordance with the reference result (Cohen’s kappa was 0.935 and 0.958 for the VIDAS and LIAISON assays, respectively). Table 3 presents the discordant samples and their resolution by WB. Most of the false-positive results obtained with the LIAISON assay were associated with low index values close to the positivity threshold (index values of approximately 1–3). Of the 13 false-negative results obtained with the VIDAS assay, eight were detected by the LIAISON assay with low antibody levels (index values between 1 and 3) (Table 3 and Fig. 1). Based on maximizing the receiver operating characteristic curves, increasing the LIAISON assay threshold to 3.04 would optimize specificity to 99.5% while maintaining sensitivity at 96.1%. For the VIDAS assay, adjusting the HSV-1 IgG threshold would not significantly change the analytical performance (Fig. S2). Analysis of the results based on one sample per patient (348 samples) also showed that the LIAISON HSV-1 IgG assay had higher sensitivity but lower specificity than the VIDAS assay (Table S2)

**TABLE 1:** Performance of the VIDAS HSV 1&2 IgG prototype and LIAISON HSV-1 IgG assays for the determination of HSV-1 IgG^*a*^

		HSV-1 IgG					
		VIDAS prototype assay		LIAISON assay			*P* value
		Pos	Neg	Pos	Neg	Eqv	
Final conclusion	Pos	270	13	280	1	2	
	Neg	3	212	9	204	2	
Sensitivity (95% CI)		95.4 (92.3–97.3)		**98.9** (96.9–99.7)			0.011
Specificity (95% CI)		**98.6** (96.0–99.5)		94.9 (91.1–97.2)			0.030
PPV (95% CI)		**99.0** (97.2–99.7)		96.7 (94.2–98.1)			0.004
NPV (95% CI)		93.5 (89.3–96.1)		**98.3** (95.2–99.4)			0.002
Cohen’s Kappa (95%CI)		0.935 (0.903–0.966)		0.942 (0.913–0.972)			0.70

^*a*^ Pos, positive; Neg, negative; Eqv, equivocal, Ind, indeterminate; PPV, positive predictive value; NPV, negative predictive value. Predictive values are calculated with an HSV-1 prevalence rate of 60%. Values in bold indicate significantly higher performance.

**Fig 1:**
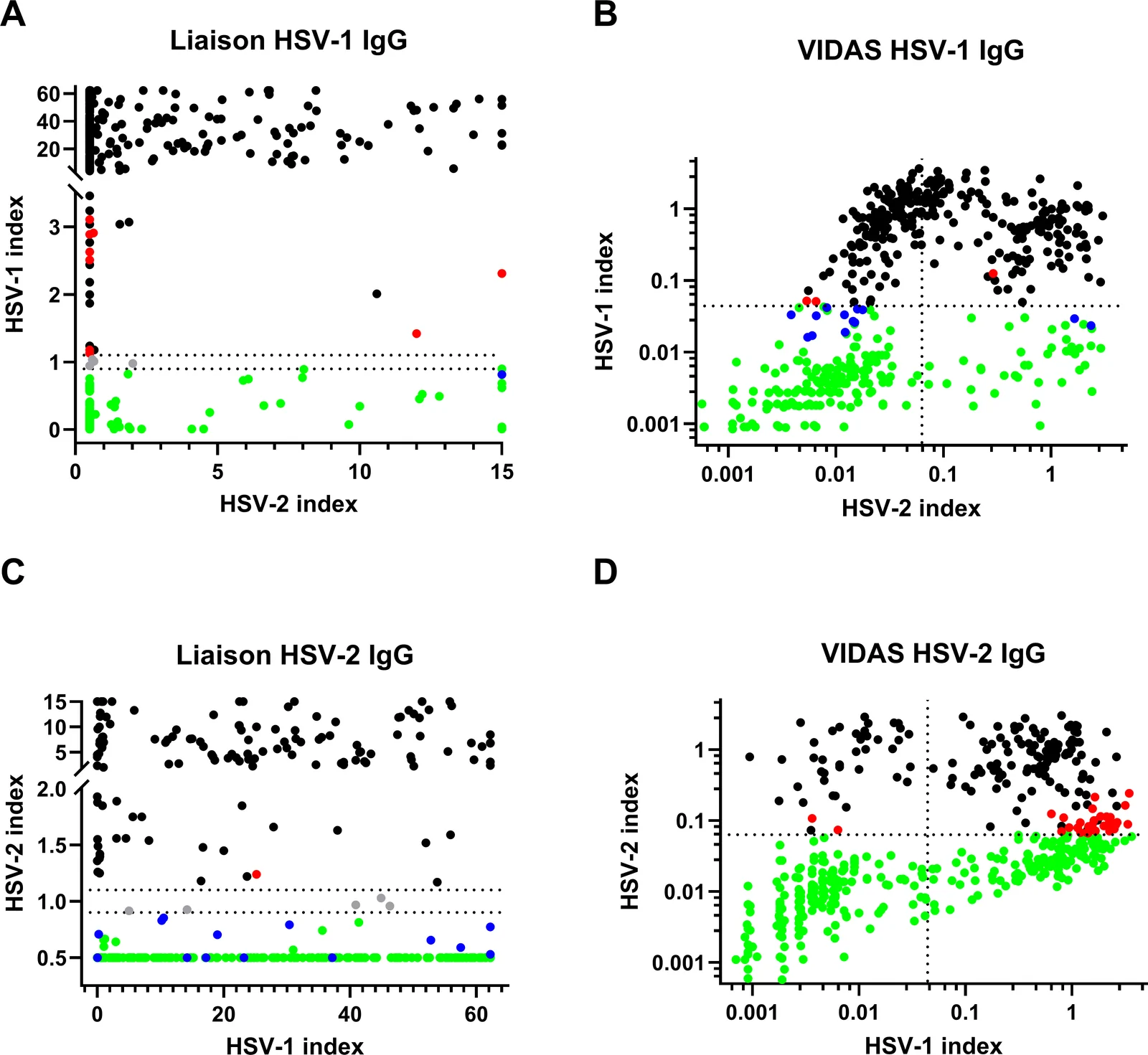
Semi-quantitative HSV-1 IgG and HSV-2 IgG assay results according to the final sample classification. True-positive samples are shown as black circles, true-negative samples as green circles, false-positive samples as red circles, false-negative samples as blue circles, and equivocal results obtained with the LIAISON assay as gray circles. (**A**) HSV-1 IgG index values obtained with the LIAISON HSV-1 IgG assay, stratified according to HSV-2 IgG index values measured with the LIAISON HSV-2 IgG assay. (**B**) HSV-2 IgG index values obtained with the LIAISON HSV-2 IgG assay, stratified according to HSV-1 IgG index values measured with the LIAISON HSV-1 IgG assay. (**C**) HSV-1 IgG index values obtained with the VIDAS HSV 1&2 IgG prototype assay, stratified according to HSV-2 IgG index values. (**D**) HSV-2 IgG index values obtained with the VIDAS HSV 1&2 IgG prototype assay, stratified according to HSV-1 IgG index values. Black dotted lines indicate the manufacturer-defined positivity thresholds for each assay.

### Assay performance for the detection of anti-HSV-2 IgG

In our cohort, for HSV-2 IgG, the sensitivity of the VIDAS HSV 1&2 IgG prototype assay was very high (100%), but its specificity was lower (90.7%). The LIAISON HSV-2 IgG assay showed lower sensitivity (88.2%) and higher specificity (99.4%) than the VIDAS assay (the analysis performed using one sample per patient yielded the same conclusions as the primary analysis; Table S2). Substantial agreement with the reference results was observed for both assays (Cohen’s kappa was 0.857 and 0.902 for the VIDAS and LIAISON assays, respectively; Table 2). The VIDAS assay yielded 32 false-positive results, corresponding to 32 samples from 24 patients. The final HSV status was established by WB analysis for 26 of these samples, whereas the remaining six required both WB findings and clinical data because the WB showed low-intensity bands. The LIAISON assay produced one false-positive result and 16 false-negative results. None of the samples that were false positives with the VIDAS assay were detected by the LIAISON assay (Table 3 and Fig. 1). The majority of the false-positive HSV-2 IgG results (30 out of 32 samples) had low index values and were associated with concomitant HSV-1 IgG detection with high index values. Among samples with high HSV-1 IgG index values (index >1, approximately 23-fold above the threshold), 30% (26/89 samples) yielded false-positive HSV-2 IgG results on the VIDAS assay (Fig. 1D). Due to the low prevalence of HSV-2 in the French population, one in three positive results on the VIDAS assay could be false positives (Table 2 ). Adjusting the threshold for the VIDAS assay based on ROC curve analysis (Fig. 2) increased the specificity of HSV-2 IgG detection to 98.8%, while maintaining a sensitivity of 95.4% at a threshold of 0.137. Decreasing the LIAISON assay threshold to 0.529 would improve sensitivity (96.7%), with a slight decrease in specificity (97.4%) (Fig. S3).

**TABLE 2:** Performance of the VIDAS HSV 1&2 IgG prototype and LIAISON HSV-2 IgG assays for the determination of HSV-2 IgG^*a*^

		HSV-2 IgG					
		VIDAS prototype assay		LIAISON assay			*P* value
		Pos	Neg	Pos	Neg	Eqv	
Final conclusion	Pos	152	0	134	14	4	
	Neg	32	313	1	343	1	
Sensitivity (95% CI)		**100** (97.5–100)		88.2 (82.1–92.6)			<0.0001
Specificity (95% CI)		90.7 (87.2–93.4)		**99.4** (97.9–99.8)			<0.0001
PPV (95% CI)		65.5 (57.3–72.6)		**96.4** (87.4–99.0)			<0.0001
NPV (95% CI)		**100** (99.5–100)		97.9 (96.9–98.7)			0.018
Cohen’s Kappa (95% CI)		0.857 (0.809–0.905)		0.902 (0.860–0.944)			0.069

^*a*^ Pos, positive; Neg, negative; Eqv, equivocal; Ind, indeterminate; PPV, positive predictive value; NPV, negative predictive value. Predictive values are calculated with HSV-2 prevalence rate of 15%. Values in bold indicate significantly higher performance.

**TABLE 3:** Discordant HSV-1 and HSV-2 IgG results between the VIDAS prototype and LIAISON assays investigated by western blot^*a*^

	HSV-1 IgG					HSV-2 IgG				
	False positives			False negatives		False positives			False negatives	
	WB profile			WB profile		WB profile			WB profile	
	−/−	−/+	Eqv^*b*^/−	+/−	+/+	−/−	+/−	+/Eqv^*b*^	−/+	+/+
LIAISON assay^*c*^	7	8	1	2	1	0	2	0	2	16
VIDAS prototype assay^*d*^	2	0	0	11	2	2	24	6	0	0
LIAISON index (min–max)	0.945–3.11			0.815–1.04		1.24			<0.5–0.957	
VIDAS index (min–max)	0.0508–0.1248			0.016–0.041		0.066–0.242			NA	

^*a*^ Data are presented separately for each WB category with the HSV-1 WB result left of the “/” and the HSV-2 WB result right of the slash. NA: not available.

^*b*^ Equivocal WB results (*n* = 7), characterized by low-intensity bands, were resolved using complementary approaches (PCR, clinical evaluation, or serological follow-up). An additional seven samples yielding equivocal WB results could not be resolved by these complementary methods and were therefore classified as indeterminate (WB profiles: equivocal/positive, *n* = 2; equivocal/negative, *n* = 1; negative/equivocal, *n* = 2; positive/equivocal, *n* = 2) (data not shown).

^*c*^ Data include equivocal results with LIAISON assays.

^*d*^ Among the 13 VIDAS HSV-1 false-negative samples, 8 were detected by the LIAISON assay, with low index values (1–3). Among the 32 VIDAS HSV-2 false-positive samples (corresponding to 24 patients), 31 were negative with the LIAISON assay (index values < 0.5), while one sample was equivocal (index 0.969).

**Fig 2:**
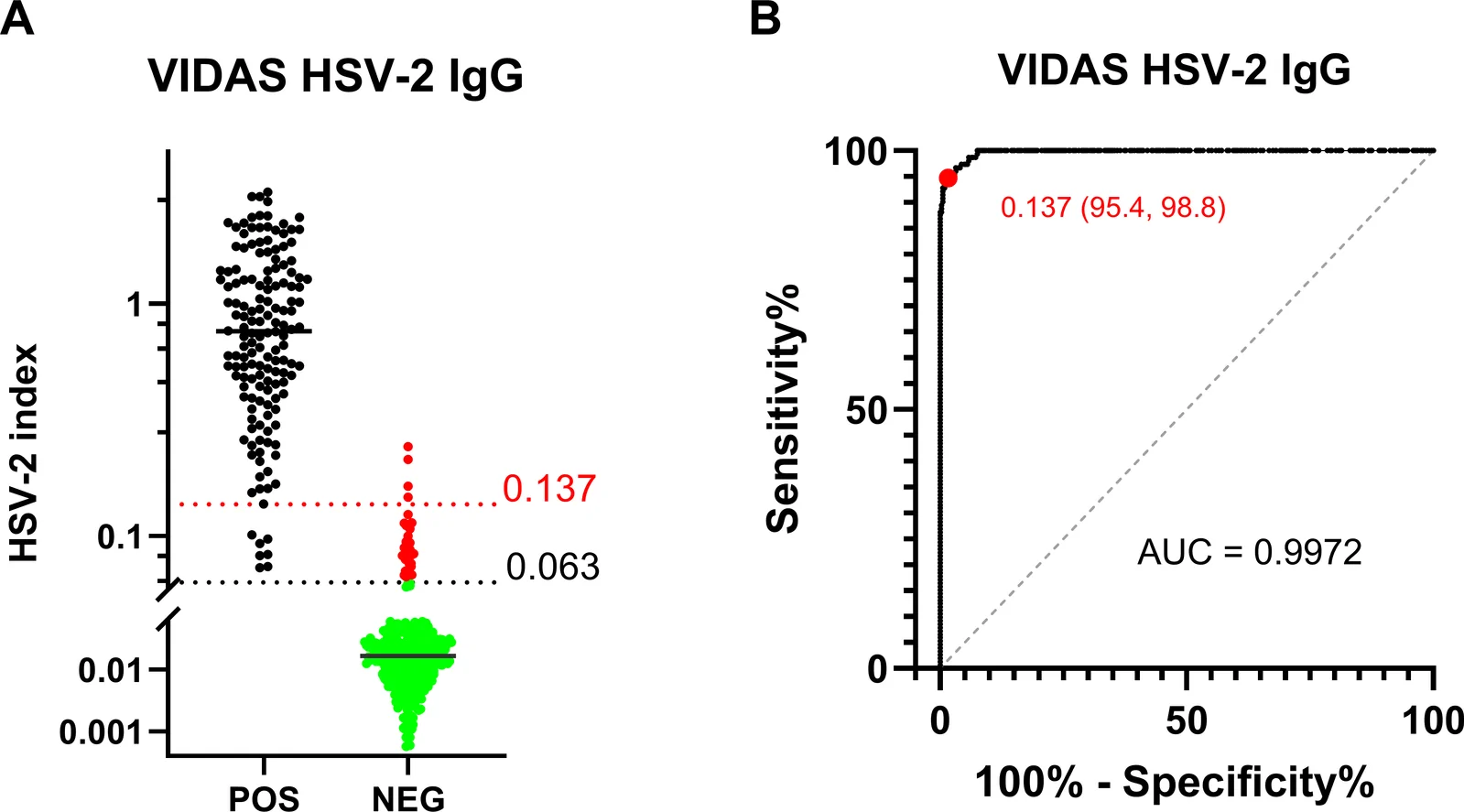
Optimization of the HSV-2 IgG positivity threshold for the VIDAS HSV 1&2 IgG prototype assay. (**A**) Distribution of HSV-2 IgG index values according to the final HSV-2 IgG classification (POS, positive; NEG, negative). True-positive samples are shown as black circles, true-negative samples as green circles, and false-positive samples as red circles. Horizontal lines indicate the median index values. Black dotted lines represent the manufacturer-defined positivity threshold, whereas orange dotted lines indicate the optimized positivity threshold. (**B**) ROC curve for HSV-2 IgG detection. The red point indicates the optimal cutoff determined using the Youden index. An optimized cutoff of 0.137 yielded a sensitivity of 95.7% and a specificity of 98.8%. AUC, area under the curve.

## DISCUSSION

Our study assessed the VIDAS HSV 1&2 IgG assay at the prototype stage, prior to potential commercialization, and compared its performance with the widely used automated LIAISON immunoassay. Both assays demonstrated comparable performance for HSV-1 IgG detection, with sensitivity and specificity ranging from 95% to 99%. However, differences were observed in HSV-2 IgG detection: the VIDAS assay showed very high sensitivity (approaching 100%) and lower specificity (90.7%) in our sample cohort, whereas the LIAISON assay exhibited high specificity (99.4%) and lower sensitivity (88.2%).

When comparing the technical characteristics of the two serological assays, several differences should be considered. The VIDAS HSV 1&2 IgG prototype assay offers the advantage of enabling simultaneous detection of anti-HSV-1 and anti-HSV-2 IgG antibodies from a single sample using a single test unit (a “biplex” assay), whereas the LIAISON assay requires two separate reagents to perform type-specific HSV IgG serology. Both assays are designed for automated testing; however, at the time of our evaluation, the prototype VIDAS assay was only available on a semi-automated instrument in our laboratory. The LIAISON assay was performed on a high-throughput, random-access platform, whereas the VIDAS assay is primarily designed for flexible, on-demand testing using single-test reagents. The VIDAS assay is characterized by its ease of use and ready-to-use reagent format. However, it requires a larger sample volume than the LIAISON assay: 100 µL to simultaneously test for HSV-1 and HSV-2 IgG, compared with 20 µL for a single marker (either HSV-1 IgG or HSV-2 IgG) using the LIAISON assay.

The study cohort comprised 501 samples from 348 immunocompetent adult patients. Most positive results (81%) were obtained from patients with PCR-confirmed active HSV-1 or HSV-2 infection. After testing all samples with both assays and resolving discordant results using WB and clinical data, we determined that 34% of samples were positive solely for anti-HSV-1 IgG, 7% solely for anti-HSV-2 IgG, 23% for both anti-HSV-1 and anti–HSV-2 IgG, and 36% negative for both. In comparison, French epidemiological studies report HSV-1 and HSV-2 IgG seroprevalence rates of 60%–65% and 12%–17%, respectively (9). Although HSV-2 prevalence is relatively low in France, anti-HSV-2 IgG-positive samples accounted deliberately for 30% of our cohort, thereby increasing the comparative data on the detection of anti-HSV-2 IgG between the VIDAS and LIAISON assays.

Comparison with previous reports revealed similar trends in the evaluation of LIAISON HSV assays. Prior studies reported HSV-1 IgG sensitivity ranging from 90% to 98%, with lower specificity (87%–91%). In contrast, HSV-2 IgG assays showed more variable sensitivity (77%–98%) depending on the cohort, but consistently higher specificity (93%–99%) than HSV-1 assays (17, 19). In our study, HSV-1 IgG sensitivity was higher than that reported by Crawford et al. (17). For the LIAISON HSV-2 IgG assay, we observed excellent specificity (99.4%), with substantially fewer false-positive results among HSV-1 IgG-positive samples than previously reported (17). However, the low sensitivity of the HSV-2 IgG assay would warrant adjusting the cutoff value if the assay were to be used for routine screening. These differences in performance may be due to variations in cohort characteristics, study design (including confirmation methods), or sample quality (e.g., storage duration). A key difference is that our study used the new, improved version of the HSV-2 IgG assay, released after December 2023, whereas Crawford et al. evaluated the previous version. This difference may also account for the discrepancies between our findings and theirs.

The VIDAS prototype assay demonstrated good overall performance for HSV-1 IgG results, comparable to that of other commercially available assays (Table S3) (17, 19). False-negative results occurred primarily in samples with low anti-HSV-1 IgG levels detectable by the LIAISON assay. However, a clear trade-off between sensitivity and specificity was observed for the VIDAS HSV-2 IgG assay. In our hands, HSV-2 IgG specificity for VIDAS was estimated at 90.7%, corresponding to 32 false-positive samples from 24 patients. The reduced specificity may be attributable to cohort characteristics, including a high proportion of patients with PCR-confirmed HSV-1 infection and a substantial number of samples with markedly elevated HSV-1 IgG levels (median index values 15–20 times above the assay positivity threshold). Consistent with previous reports (7, 17), false-positive HSV-2 IgG results were associated with low index values (a pattern also observed in our study) and occurred more frequently in samples positive for HSV-1. Notably, 30% of samples with high HSV-1 IgG index values in our cohort (index >1, approximately 23-fold above the threshold) yielded false-positive HSV-2 IgG results with the VIDAS assay. Note that one patient with HSV-1 reactivation and high HSV-1 IgG levels contributed four false-positive samples, suggesting also interindividual variability in anti-HSV-1 antibody responses and potential interference with the HSV-2 IgG assay.

We further evaluated whether adjusting the index threshold could improve assay performance. ROC curve analysis indicated that increasing the VIDAS HSV-2 IgG threshold from 0.063 to 0.137 would improve specificity to 98.8% (reduction from 32 to 4 false positives) while maintaining sensitivity at 95.4%. Such adjustment is necessary to reduce false-positive rates, particularly in populations with low HSV-2 prevalence. Type-specific HSV serology is recommended in certain clinical contexts, such as in pregnant women with genital herpes, where the results influence neonatal management (11). For instance, in a pregnant woman with a PCR-confirmed HSV-2 genital infection at delivery, a false-positive HSV-2 IgG result could lead to incorrect classification as recurrent infection, resulting in inappropriate clinical reassurance. These findings highlight the importance of high assay specificity for HSV-1 or HSV-2 IgG detection and discrimination.

This study has several limitations. Assay performance estimates depend on cohort characteristics, and our cohort included a relatively high proportion of challenging samples (23% dual HSV-1/HSV-2 IgG positivity), particularly affecting specificity assessment. In the absence of a reference method applied to all samples, concordant false-positive or false-negative results cannot be excluded, potentially leading to overestimation of assay performance. Repeat testing was not performed for samples with index values near assay cutoffs. Finally, the VIDAS assay evaluated here is a prototype, and performance characteristics may differ in future commercial versions. Nevertheless, our independent evaluation provides data that may help guide assay optimization. Prospective studies, particularly in specific populations such as pregnant women, are warranted to validate optimized thresholds, especially for HSV-2 IgG detection.

In conclusion, the VIDAS HSV 1&2 IgG prototype assay performs comparably to the LIAISON assay for HSV-1 IgG detection. For HSV-2 IgG detection, adjusting the positivity threshold may significantly enhance clinical utility, particularly in low-prevalence settings. Although the overall performance of currently available commercial HSV-1 and HSV-2 immunoassays remains imperfect (especially regarding HSV-1 sensitivity and HSV-2 specificity), the VIDAS prototype biplex assay, as well as its likely optimized future version, demonstrated overall satisfactory performance in our hands and may be useful in the clinical management of patients with herpes infection.

## SUPPLEMENTAL MATERIAL

Fig. S1 to S3 jcm.00931-26-s0001.docx Design of the study and optimization of positivity thresholds for the Vidas and Liaison HSV-1 IgG assays, and the Liaison HSV-2 IgG assay.

Tables S1 to S3 jcm.00931-26-s0002.xlsx Characteristics of the patient, performance of the assays using one sample per patient, and comparison of the assays in previous studies.

ASM does not own the copyrights to Supplemental Material that may be linked to, or accessed through, an article. The authors have granted ASM a non-exclusive, world-wide license to publish the Supplemental Material files. Please contact the corresponding author directly for reuse.
