# Supplementary material for: Evaluation of a prototype biplex VIDAS immunoassay for type-specific detection of HSV-1 and HSV-2 IgG antibodies
Source: J Clin Microbiol. 2026 Sep 30;64(11):e00931-26. doi: 10.1128/jcm.00931-26 (PMC13753811; doi:10.1128/jcm.00931-26)
Supplement: Supplementary file 1 [file jcm.00931-26-s0001.docx]

**Supplemental Figures**

**Figure S1**: Flow chart of the study

**Figure S2.** Optimization of the positivity thresholds for the Liaison and VIDAS® HSV-1 IgG assays. (A) Distribution of Liaison HSV-1 IgG index values according to the final HSV-1 IgG classification (POS, positive; NEG, negative). (B) Receiver operating characteristic (ROC) curve for the Liaison HSV-1 IgG assay. The red point indicates the optimal cutoff determined using the Youden index. An optimized cutoff of 3.04 yielded a sensitivity of 96.1% and a specificity of 99.5%. (C) Distribution of VIDAS^®^ HSV-1 IgG index values according to the final HSV-1 IgG classification (POS, positive; NEG, negative). (D) Receiver operating characteristic (ROC) curve for the VIDAS^®^ HSV-1 IgG prototype assay. The red point indicates the optimal cutoff determined using the Youden index. An optimized cutoff of 0.032 yielded a sensitivity of 97.5% and a specificity of 96.7%. AUC, area under the curve. True-positive samples are shown as black circles, true-negative samples as green circles, false-positive samples as red circles, false-negative samples as blue circles, and equivocal results obtained with the Liaison assay as grey circles. Horizontal lines indicate the median index values. Black dotted lines represent the manufacturer-defined positivity thresholds, whereas orange dotted lines indicate the optimized positivity thresholds.

**Figure S3.** Optimization of the positivity threshold for the Liaison HSV-2 IgG assay. (A) Distribution of Liaison HSV-2 IgG index values according to the final HSV-2 IgG classification (POS, positive; NEG, negative). (B) Receiver operating characteristic (ROC) curve for the Liaison HSV-2 IgG assay. The red point indicates the optimal cutoff determined using the Youden index. An optimized cutoff of 0.529 yielded a sensitivity of 96.7% and a specificity of 97.4%. AUC, area under the curve. True-positive samples are shown as black circles, true-negative samples as green circles, false-positive samples as red circles, false-negative samples as blue circles, and equivocal results obtained with the Liaison assay as grey circles. Horizontal lines indicate the median index values. Black dotted lines represent the manufacturer-defined positivity threshold, whereas orange dotted lines indicate the optimized positivity threshold.
